# Investigations of the Contribution of a Putative Glycine Hinge to Ryanodine Receptor Channel Gating*

**DOI:** 10.1074/jbc.M113.465310

**Published:** 2013-04-30

**Authors:** Joanne Euden, Sammy A. Mason, Cedric Viero, N. Lowri Thomas, Alan J. Williams

**Affiliations:** From the Institute of Molecular and Experimental Medicine, Cardiff University, Cardiff CF14 4XN, Wales, United Kingdom

**Keywords:** Calcium Channels, Calcium Intracellular Release, Ion Channels, Ryanodine Receptor, Sarcoplasmic Reticulum (SR), Glycine Hinge, Single Channel Gating

## Abstract

Ryanodine receptor channels (RyR) are key components of striated muscle excitation-contraction coupling, and alterations in their function underlie both inherited and acquired disease. A full understanding of the disease process will require a detailed knowledge of the mechanisms and structures involved in RyR function. Unfortunately, high-resolution structural data, such as exist for K^+^-selective channels, are not available for RyR. In the absence of these data, we have used modeling to identify similarities in the structural elements of K^+^ channel pore-forming regions and postulated equivalent regions of RyR. This has identified a sequence of residues in the cytosolic cavity-lining transmembrane helix of RyR (G^4864^LIIDA^4869^ in RyR2) analogous to the glycine hinge motif present in many K^+^ channels. Gating in these K^+^ channels can be disrupted by substitution of residues for the hinge glycine. We investigated the involvement of glycine 4864 in RyR2 gating by monitoring properties of recombinant human RyR2 channels in which this glycine is replaced by residues that alter gating in K^+^ channels. Our data demonstrate that introducing alanine at position 4864 produces no significant change in RyR2 function. In contrast, function is altered when glycine 4864 is replaced by either valine or proline, the former preventing channel opening and the latter modifying both ion translocation and gating. Our studies reveal novel information on the structural basis of RyR gating, identifying both similarities with, and differences from, K^+^ channels. Glycine 4864 is not absolutely required for channel gating, but some flexibility at this point in the cavity-lining transmembrane helix is necessary for normal RyR function.

## Teaser

**Background:** In many K^+^ channels, gating requires flexing of inner helices at glycine residues within a “hinge motif.” An analogous motif is present in the ryanodine receptor channel.

**Results:** Mutation of ryanodine receptor “hinge” glycines produces varying effects on gating.

**Conclusion:** Ryanodine receptors do not contain a typical glycine hinge.

**Significance:** This work provides new insights into mechanisms of ryanodine receptor function.

## Introduction

The cardiac ryanodine receptor (RyR2)^3^ is a large intracellular ion channel that provides the pathway for the regulated release of stored calcium from the lumen of the sarcoplasmic reticulum into the cytoplasm, initiating muscle contraction in response to cell excitation (1). Mutations within RyR2 are established as the underlying cause of the inherited disease catecholaminergic polymorphic ventricular tachycardia type 1 (CPVT1) (2, 3); however, the mechanisms that lead to altered function are yet to be fully established. To gain an insight into how function is altered in mutant channels, we need greater knowledge of the structures involved in the ion permeation pathway and the mechanisms whereby transitions between open and nonconducting conformations occur. Unfortunately, at present, no high-resolution structural information is available for the pore-forming region (PFR) of the RyR channel.

**FIGURE 1.**
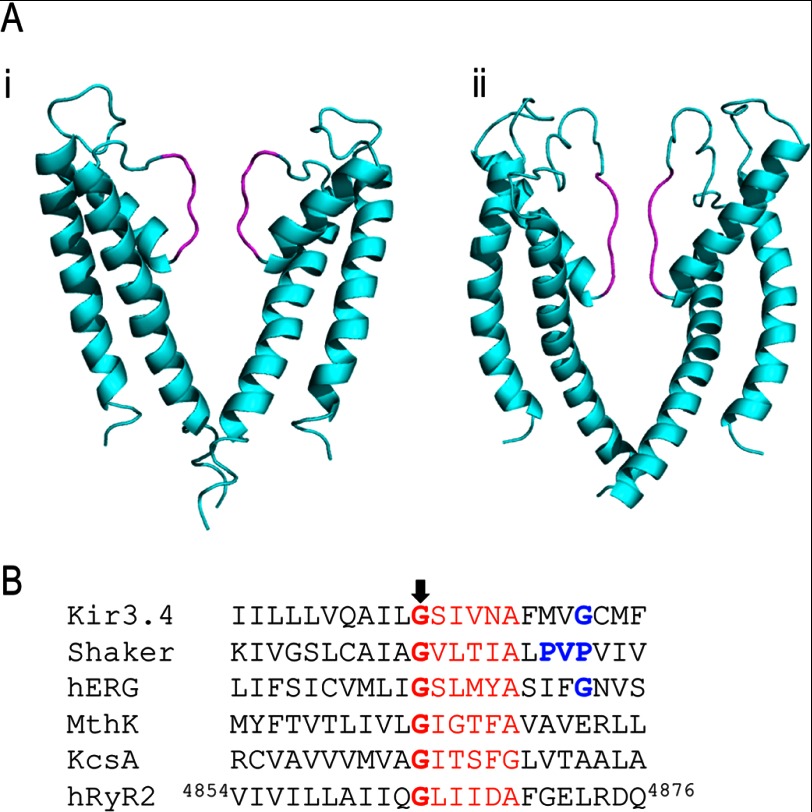
**Comparison of KcsA and the RyR2 analogy model and sequence alignment of the inner helices of a range of K^+^ channels with the human RyR2 sequence.** *A*, PyMOL (34) view of the RyR2 PFR. The panel depicts a comparison of the structural elements of KcsA (*panel i*) and our RyR2 pore analogy model (*panel ii*), constructed using KcsA as a template. Two monomers are shown for clarity. The main chain polypeptide backbone is colored in *cyan*, whereas the selectivity filter is highlighted in *magenta*. The luminal side of the pore appears at the *top. B*, the glycine residue of the conserved hinge motif is highlighted, in all cases, in *bold red* with the other motif residues in *red*. Additional residues and motifs involved in gating are highlighted in *bold blue*.

Previous work from our group, involving the construction of an analogy model based upon the known structure of the bacterial K^+^ channel KcsA (4), indicates that the PFR of RyR2 is composed of structural elements equivalent to those found in K^+^ channels and that these elements are likely to have a similar topology (Fig. 1*A*). This conclusion was reinforced by investigations employing molecular modeling (5) and cryo-electron microscopy (6, 7) to study the PFR of the skeletal isoform of the RyR channel (RyR1). In the absence of structural data at atomic resolution, RyR PFR models provide us with frameworks that can be used to predict and test the involvement of domains and individual residues in the processes of ion translocation (8) and gating (9).

Crystal structures of K^+^- and Na^+^-selective channels have greatly enhanced our understanding of the mechanisms underlying channel function. The structure of KcsA, corresponding to the closed conformation, shows all four inner helices of the PFR to be straight, coming together at the intracellular entrance of the pore to form a hydrophobic constriction (10). In comparison, the MthK structure, crystallized with its bound physiologically activating ligand (Ca^2+^), is thought to represent an open structure and shows the inner helices splaying outwards at the middle of the cytosolic cavity-lining helices with flexing occurring at conserved glycine residues located just below the selectivity filter (11, 12).

These conserved glycine residues occur as part of a G*XXXX*(A/G) motif seen in KcsA, MthK, and many other ligand- and voltage-gated K^+^ channels (Fig. 1*B*) (12–15). In some K^+^ channels, an additional glycine occurs nine residues downstream, as in the case of inward rectifying channels (16) and hERG (17), or a PVP motif is located seven residues downstream, as in the case of *Shaker* voltage-gated K^+^ channels (18) (Fig. 1*B*). These regions have been proposed to introduce either an alternative site for a hinge or a pronounced kink in the helix, forming a site of weakness, which could contribute to channel gating. Highly conserved sequence similarities strongly suggest that common gating mechanisms exist in K^+^ channels irrespective of the opening stimulus.

The RyR2 analogy model identifies transmembrane helix 10 (TM10) as lining the cytosolic cavity of the PFR and equivalent to the inner helices of K^+^ channels (19). Studies using recombinant mouse RyR2 have demonstrated that mutation of residues in this helix influences the interaction of both blocking molecules (20) and ryanodine (21, 22) with the channel and can modify the response to activating ligands (23). Importantly, the RyR2 PFR model also identifies a sequence of residues in TM10 (G^4864^LIIDA^4869^) that is equivalent to the G*XXXX*A hinge motif identified in K^+^ channels (4), and this hinge motif is also present in RyR1 TM10 (5, 6). RyR TM10s do not contain the additional conserved glycine residue found in the inward rectifiers or a PVP motif (Fig. 1*B*) (4).

In voltage-sensitive K^+^ channels, the activation signal is transmitted from the voltage-sensing domain to the inner helices of the channel via interactions between residues of a short helix that links transmembrane helices 4 and 5 (S4-S5 linker) and residues of the inner helix (S6) at the cytosolic entrance to the PFR (24–26). A very recent investigation has identified an equivalent mechanism in RyR1 and has demonstrated that mutations that alter interactions between residues in the linking and inner helices of RyR1 can produce significant changes in channel gating (9). In the model presented by Ramachandran *et al.* (9), ligand-mediated transitions between closed and open conformations of RyR1 are proposed to occur as the result of flexing at glycine 4934. This residue is equivalent to glycine 4864 in RyR2 and is the putative glycine hinge identified in the G^4864^LIIDA^4869^ motif of the inner helix of the RyR2 PFR model (4).

In the current study, we describe the first experimental evaluation of the contribution of a potential glycine hinge to RyR channel gating by monitoring the function of recombinant human RyR2 channels in which glycine 4864 has been replaced by alanine, valine, or proline. The novel data arising from this study establish that a degree of flexibility at position 4864 in the helices that are proposed to line the cytosolic cavity of the PFR is required to allow transitions between closed and open conformations of RyR. A comparison of the results obtained in this study with data from earlier studies in K^+^ channels highlights both similarities and differences in the roles played by glycine hinge residues in these different species of channel.

## EXPERIMENTAL PROCEDURES

### 

#### 

##### Materials

Caffeine and standard chemicals, at the best available grade, were obtained from Sigma-Aldrich (Poole, Dorset, UK). [^3^H]Ryanodine was from PerkinElmer Life Sciences. Synthetic 1-palmitoyl-2-oleoyl-*sn*-glycero-3-phosphoethanolamine was supplied by Avanti Polar Lipids (Alabaster, AL).

##### Site-directed Mutagenesis, Expression of Wild Type and Mutant cDNAs in HEK Cells, and Membrane and Channel Purification

Point mutations were introduced into TM10 (14) of full-length, N-terminally enhanced green fluorescent protein (eGFP)-tagged, human RyR2 cDNA at position Gly^4864^ by oligonucleotide-directed mutagenesis as described previously (27). Oligonucleotides were reverse-phase purified (Sigma-Genosys, Cambridge, UK), and their sequences are as follows: G^4864^AF, CATTCTCTTGGCCATAATACAAGCTCTAATTATTGATGCTTTTGGAG; G^4864^AR, CTCCAAAAGCATCAATAATTAGACCTTGTATTATGGCCAAGAGAATG; G^4864^VF, CATTCTCTTGGCCATAATACAAGTTCTAATTATTGATGCTTTTGGAG; G^4864^VR, CTCCAAAAGCATCAATAATTAGAACTTGTATTATGGCCAAGAGAATG; G^4864^PF, CATTCTCTTGGCCATAATACAACCTCTAATTATTGATGCTTTTGGAG; and G^4864^PR, CTCCAAAAGCATCAATAATTAGAGGTTGTATTATGGCCAAGAGAATG.

All constructs were verified by automated sequencing (ABI 3700, Applied Biosystems). Full-length WT and mutant cDNAs were propagated in XL10 Gold cells (Stratagene) following stringent procedures, and large scale plasmid purification was performed using a gel-based purification system (Qiagen). High purity WT or mutant plasmid cDNA (12 μg) was transfected into human embryonic kidney (HEK) cells using a calcium phosphate precipitation method. Prior to transfection, HEK293 cells were grown in supplemented Dulbecco's modified Eagle's medium (DMEM; Invitrogen, Paisley, Renfrewshire, UK) for 18 h in 100-mm diameter tissue culture dishes seeded at a density of 1.5 × 10^6^ cells/dish. Expression of recombinant protein in the endoplasmic reticulum was verified 24 h after transfection by inspection of the fluorescence level of the eGFP tag attached at the N terminus of the full-length RyR2 cDNA sequence (28).

Mixed membrane populations were isolated following cell homogenization in ice-cold buffer (20 mm Tris, 1 mm EDTA, pH 7.4) in the presence of a serine/cysteine protease inhibitor mixture (Roche Diagnostics Ltd., West Sussex, UK). The homogenate was centrifuged at 1700 × *g* for 15 min at 4 °C to remove cell debris, and the resultant supernatant was centrifuged at 100,000 × *g* for 1 h at 4 °C to pellet membranes. Mixed membrane pellets were resuspended in 0.4 m sucrose, 20 mm HEPES, pH 7.4, and the protein concentration was determined with a BCA assay kit (Pierce Biotechnology) before flash freezing in liquid nitrogen and storage at −80 °C.

To purify RyR2 channels, ∼8 mg of mixed membrane protein was solubilized for 1 h at 4 °C using 0.6% CHAPS and 0.3% phosphatidylcholine at 2 mg of protein/ml. Following a low speed (14,000 × *g*) spin, the solubilized supernatant was placed on top of a continuous (0–40%) sucrose gradient and centrifuged at 100,000 × *g* for 17 h at 4 °C. Sucrose gradients were separated into 1.5-ml fractions, and the density of the fractions was assessed using a 0–50% sugar refractometer (Bellingham & Stanley Ltd., Tunbridge Wells, Kent, UK) to identify fractions at ∼28% that contain functional RyR2 channels. Aliquots from these fractions were flash-frozen in liquid nitrogen and stored at −80 °C.

##### Measurement of Intracellular Ca^2+^ Dynamics Using Confocal Microscopy

For recording intracellular Ca^2+^ dynamics, HEK293 cells were transfected using Effectene (Qiagen). Cells were seeded at 1 × 10^5^ on laminin (1 mg/ml; Sigma)-coated glass coverslips (MatTek Corp.). Transfection efficiency was evaluated by monitoring the level of the eGFP fluorescence in intact cells (usually ∼40%). Cells maintained in minimum DMEM (1.8 mm CaCl_2_, pH 7.4) were loaded with the Ca^2+^-sensitive fluorescent probe fluo3-AM (10 μm in 20% (w/v) Pluronic F-127 in dimethyl sulfoxide (DMSO) (Life Technologies)) for 1 h at 30 °C. Following de-esterification, RyR2-mediated Ca^2+^ release from the intracellular stores was initiated by the addition of caffeine (10 mm, applied as a bolus in DMEM) to cells and monitored using an SP5 confocal microscope (Leica Microsystems, Heidelberg, Germany). Fluo3 required excitation at 488 nm. Fluorescence emission was detected above 500 nm. Series of images were recorded at acquisition speeds ranging from 20 to 30 Hz. All experiments were performed against a background of comparable endoplasmic reticulum Ca^2+^ load without any stimulation or treatment prior to caffeine application. Data were acquired from regions of interest representing global Ca^2+^ environments (typically ∼50 μm^2^) and analyzed using Leica LAS AF confocal software (Leica Microsystems) and GraphPad Prism software (GraphPad Software, San Diego, CA).

##### Western Blot Analysis

Mixed membrane samples were resuspended in SDS-PAGE loading buffer (60 mm Tris base, pH to 6.8 with HCl, 2% SDS, 10% glycerol, 5 mm EDTA, 0.05% bromphenol blue), heated at 42 °C for 20 min, and centrifuged at 13,000 × *g* for 1 min before loading on 5% SDS-PAGE gels. Following separation, proteins were electrophoretically transferred onto an incorporated polyvinylidene difluoride membrane using a dry transfer system (iBlot, Invitrogen) for 13 min. The membrane was blocked with 5% nonfat milk protein in TBS-T buffer (20 mm Tris-HCl, pH 7.6, 137 mm NaCl, and 0.1% Tween 20). Primary antibody was applied for 90 min at room temperature (anti-GFP antibody at 1:5000 dilution (Santa Cruz Biotechnology, mouse monoclonal)) followed by three 5-min washes in TBS-Tween. Secondary antibody was added at 1:5000 dilution as above (sheep anti-mouse HRP). Immunoreactive protein bands were visualized by enhanced chemiluminescence detection (Pierce ECL, Thermo Scientific).

##### [^3^H]Ryanodine Binding Assay

The ability of WT or mutant RyR2 to bind ryanodine was monitored using mixed membrane populations isolated from HEK293 cells. As the level of expression can vary between preparations, the RyR2 content of each mixed membrane population was determined by densitometry following Western blot analysis as described above. In each binding assay, the quantity of mixed membranes expressing mutant RyR2 was adjusted to ensure that the quantity of RyR2 was equivalent to that present in 100 μg of protein of the accompanying WT mixed membrane sample. Membranes were incubated with 10 nm [^3^H]ryanodine at 37 °C for 90 min with constant shaking in a buffer medium, designed to optimize channel open probability (1 m KCl, 100 μm total Ca^2+^, and 25 mm PIPES, pH 7.4). All assays were performed in triplicate, and the number of separate mixed membrane preparations is detailed under “Results.” Nonspecific binding was determined in the presence of a 1000-fold excess (10 μm) of unlabeled ryanodine. Binding was terminated by the addition of 5 ml of buffer medium followed immediately by vacuum filtration through Whatman GF/F filters presoaked in buffer medium. To remove residual unbound [^3^H]ryanodine, the filters were washed with a further two aliquots of buffer medium. Radioactivity remaining on the filter was determined by placing the filter in 10 ml of aqueous Optima Gold MV scintillant mixture (Packard BioScience). Vials were vortexed and left to soak for at least 30 min to allow the filters to equilibrate with the scintillant before [^3^H]ryanodine was quantified by liquid scintillation counting. Specific [^3^H]ryanodine binding was calculated from total by subtracting nonspecific binding.

##### Single Channel Recording in Planar Phospholipid Bilayers

Planar phospholipid bilayers were formed from a suspension of synthetic 1-palmitoyl-2-oleoyl-*sn*-glycero-3-phosphoethanolamine in *n*-decane (35 mg/ml) by painting across a 200-μm-diameter hole in a partition separating the *cis* (0.5-ml) and *trans* (1.0-ml) chambers. The *trans* chamber was held at ground, and the *cis* chamber was clamped at potentials relative to ground. Current flow was measured using an operational amplifier as a current-voltage converter (29, 30). Bilayers were formed in symmetrical solutions containing 200 mm KCl, 20 mm HEPES titrated to pH 7.2 with KOH, resulting in solutions containing 210 mm K^+^ in both chambers. To measure single channel currents, aliquots of RyR2-containing fractions were added to the *cis* chamber, and channels were incorporated by imposing a KCl gradient (31). Following incorporation, unincorporated channels and the osmotic gradient were removed by perfusion. RyR2 channels incorporate in a fixed orientation with the cytosolic face exposed to the *cis* chamber and the luminal face exposed to the *trans* chamber (31). Ca^2+^ dependence was tested by the *cis* addition of 1 mm EGTA, HEDTA, and nitrilotriacetic acid to lower free Ca^2+^ to a subactivating concentration (0.7 nm); channels were then activated by increasing the *cis* free Ca^2+^ 100 μm (calculated using MaxChelator software). The luminal Ca^2+^ concentration was buffered to 50 nm throughout, and all experiments were performed at room temperature (22 ± 2 °C).

##### Single Channel Data Acquisition and Analysis

Single channel current fluctuations were low pass-filtered at 5 kHz with an eight-pole Bessel filter and sampled at 20 kHz with a PCI-6036E A-D board (National Instruments, Austin, TX) for acquisition using Acquire 5.0.1 (Bruxton Corp., Seattle, WA). Single channel current amplitudes were obtained from all-point histograms using TACx4.1.5 (Bruxton Corp.). Dwell times of open and closed states, in each calcium condition, were determined by analysis of recordings of at least 2-min duration using QuB Express software (the State University of New York (SUNY), Buffalo, NY). Current fluctuations were idealized with the hidden Markov modeling algorithm of the QuB 1.5.0.39 suite employing a simple two-state scheme (Closed ⇆ Open).

##### Statistical Analysis

Results were analyzed using GraphPad Prism 5.02 (GraphPad Software). Unpaired Student's *t* tests were used for analyzing the statistical significance of changes between one experimental condition and the corresponding control. Effects were regarded as significant when *p* < 0.05 (*, *p* < 0.05, **, *p* < 0.01, ***, *p* < 0.001). The results are expressed as mean values ± S.E. Normal distributions were assessed by the Kolmogorov-Smirnov test.

## RESULTS

We have investigated the contribution of Gly^4864^ to RyR2 gating by monitoring the function of channels in which this putative hinge point has been replaced with residues likely to modify the flexibility of the cytosolic cavity-lining helices at this position.

### 

#### 

##### Expression of WT and Mutant RyR2 Channels in HEK293 Cells

**FIGURE 2.**
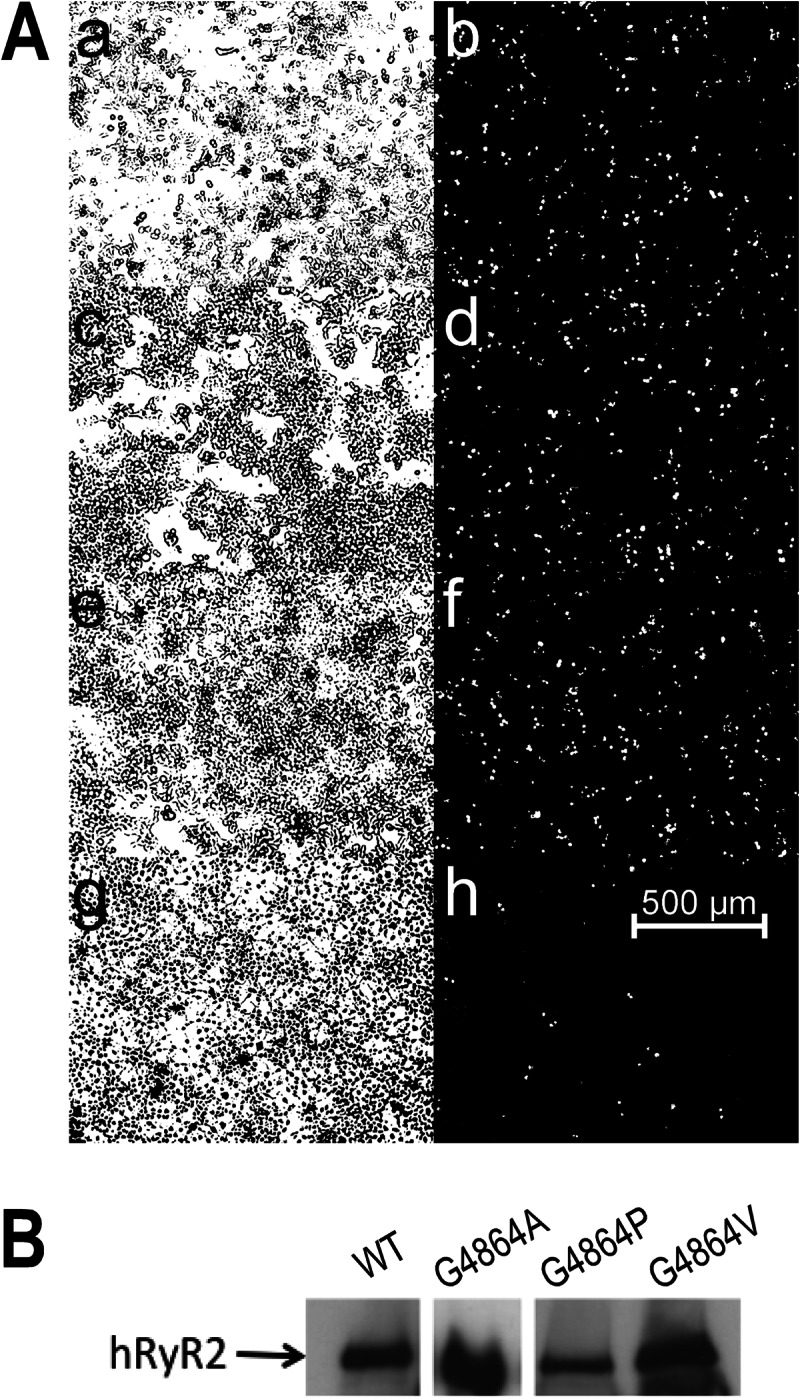
**Expression of hRyR2 proteins in HEK293 cells and Western blot analysis.** *A*, the endogenous fluorescence of eGFP-tagged hRyR2 (*right panels b*, *d*, *f*, and *h*) was used to determine the efficiency of plasmid transfection by comparison with the total number of cells seen in the phase image (*left panels a* ,*c*, *e*, and *g*). *Panels a* and *b*, WT; *panels c* and *d*, G4864A; *panels e* and *f*, G4864V; *panels g* and *h*, G4864P. *B*, this panel shows the expression of eGFP-tagged proteins in mixed membrane preparations from HEK293 cells transfected with WT, G4864A, G4864P, and G4864V hRyR2 cDNA. 100 μg of protein was loaded on each lane. Relative levels of expression were quantified by densitometry. Note that the G4864A band was obtained from a different blot.

The efficiency of transfection of WT and mutant RyR2 channels was determined by monitoring the endogenous fluorescence of the eGFP-tagged protein. Representative populations of WT and mutant RyR2-transfected cells are shown in Fig. 2*A*. The transfection efficiencies of G4864A and G4864V were equivalent to WT RyR2. G4864P was expressed in a noticeably smaller proportion of cells. Expression of RyR2 in the cells was confirmed by Western blotting of mixed membranes isolated following transfection, with representative blots for WT and mutant RyR2 shown in Fig. 2*B*.

##### Caffeine-induced Release of Stored Ca^2+^ in Intact Transfected HEK293 Cells

**FIGURE 3.**
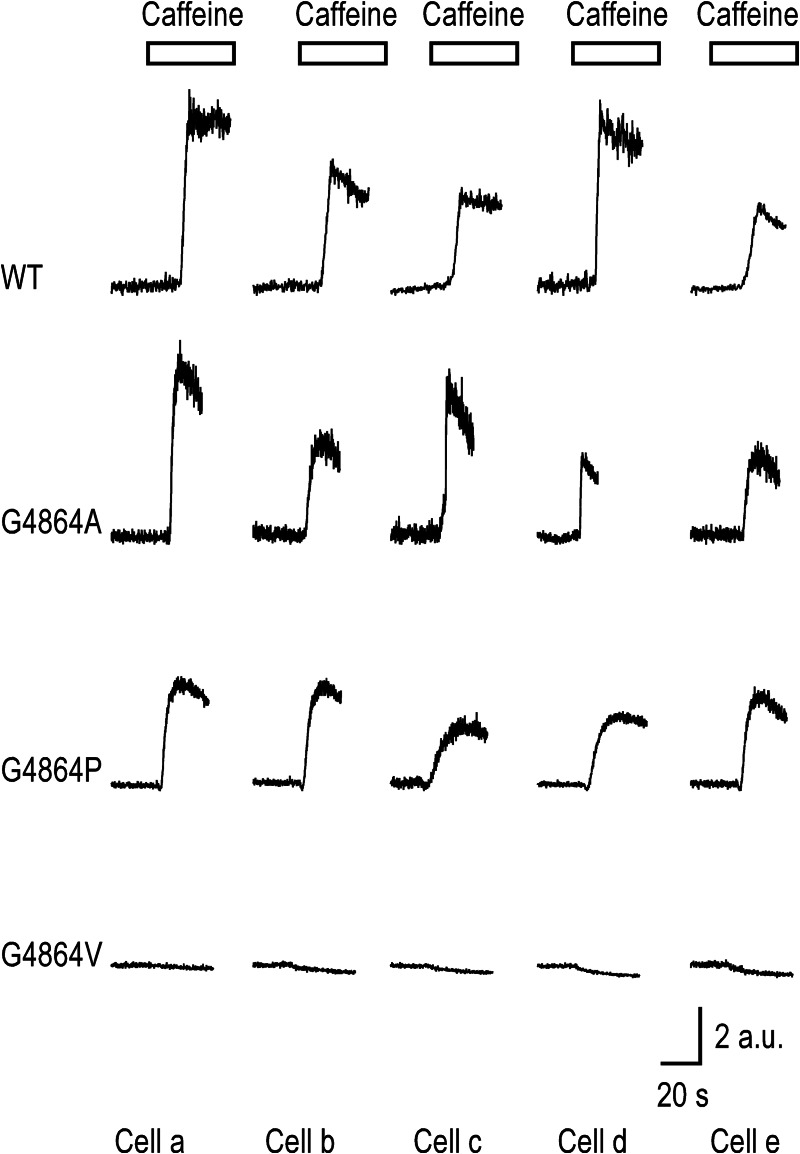
**Caffeine-induced release of Ca^2+^ from intracellular stores in HEK cells transfected with WT and mutant RyR2 channels.** Fluorescence of fluo-3-AM loaded cells was monitored continuously before and after the addition of 10 mm caffeine. Traces shown are from representative experiments that have been repeated in at least three different coverslips for three different transfections. Similar responses were measured in all experiments. These graphs depict the normalized change in fluorescence (corrected from the background) Δ*F*/*F*_0_ over time. Responses from five different cells are shown for each channel type. *a.u.*, absorbance units.

An initial qualitative assessment of the functional state of the WT and mutant RyR2 channels *in situ* was obtained by monitoring the release of Ca^2+^ from intracellular stores in response to the application of 10 mm extracellular caffeine. Fig. 3 contains representative traces showing caffeine-induced changes in intracellular Ca^2+^-dependent fluorescence. In cells transfected with WT RyR2, caffeine evoked a rapid, 4–9-fold, increase of fluorescence. Cells expressing RyR2 in which Gly^4864^ had been replaced by alanine showed caffeine-induced changes in fluorescence comparable with WT. Cells in which RyR2 Gly^4864^ was replaced by proline also released stored Ca^2+^ in response to a caffeine challenge; however, consistent with the lower transfection efficiency seen with G4846P, the proportion of caffeine-sensitive cells was lower than that seen with either WT or alanine substituted RyR2. These results demonstrate that RyR2 channels in which Gly^4864^ has been replaced by either alanine or proline can be induced to open by caffeine and release stored Ca^2+^. In contrast, cells expressing RyR2 in which Gly^4864^ has been replaced by valine showed no release of stored intracellular Ca^2+^ in response to caffeine application. The small reduction in fluorescence seen in the G4864V cells on the addition of caffeine is due to quenching by this ligand (18).

##### Ryanodine Binding Properties of WT and Gly^4864^ Mutant RyR2 Channels

**FIGURE 4.**
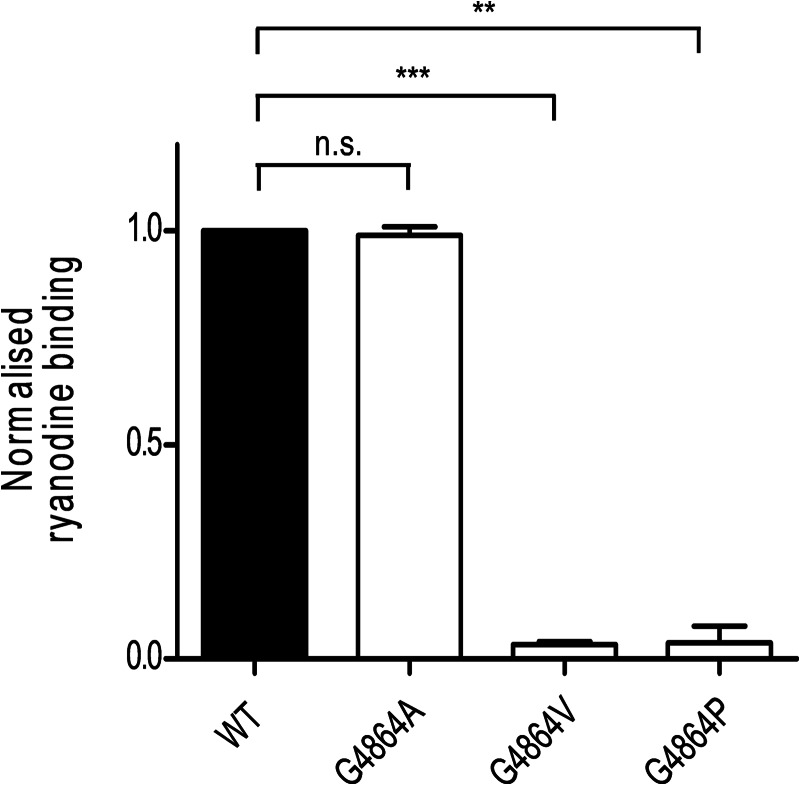
**[^3^H]Ryanodine binding to mixed membranes.** Experiments were performed in binding buffer containing 100 μm total Ca^2+^. Reactions contained 100 μg of WT protein and equivalent amounts of mutant RyR2 protein (normalized for differing levels of expression as explained under ”Results“). Data are plotted as mean ± S.E. of nine assays (three replicates from each of three membrane preparations). *n.s.*, not significant.

As the high affinity binding site for ryanodine in RyR2 is accessible only in open conformations of the channel, the functional state of populations of RyR2 channels can alternatively be assessed, and compared quantitatively, by determining the ability of membrane fractions, in which RyR2 is expressed, to bind [^3^H]ryanodine. For a valid comparison of the binding capabilities of the WT and mutant RyR2s, Western blot analysis was used to determine the relative content of RyR2 in each membrane preparation, and the total mixed membrane protein was adjusted so that an equivalent quantity of RyR2 was present in each assay. Values of specific Ca^2+^-stimulated ryanodine binding to mutant RyR2, expressed as a proportion of the binding to the WT protein, are shown in Fig. 4. This assay demonstrates that replacement of Gly^4864^ with alanine produces no significant alteration in the interaction of ryanodine with RyR2. In contrast, replacement of Gly^4864^ with either a valine or a proline residue results in a very significant reduction in the ability of RyR2 to bind ryanodine.

##### Single Channel Properties of WT and Gly^4864^ Mutant RyR2 Channels

**FIGURE 5.**
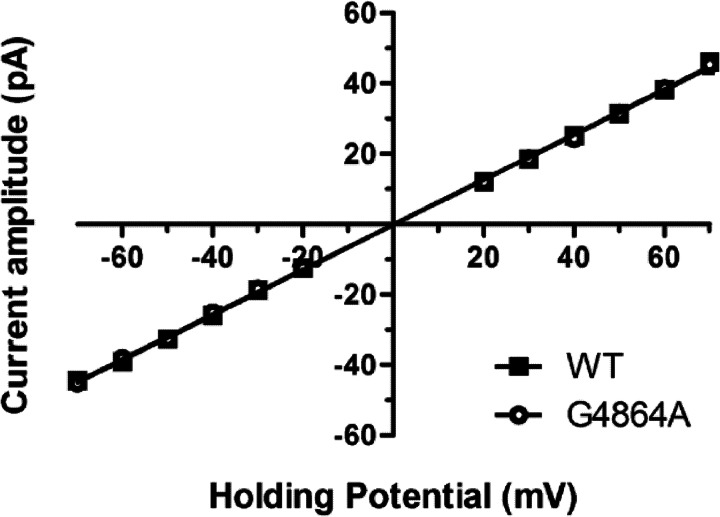
**Current-voltage relationship for WT and G4864A RyR2.** Single channel conductance was measured in 210 mm KCl, 20 mm HEPES at pH 7.2. Data are plotted as mean ± S.E. For WT, *n* = 5 or 6 individual channels. For G4864A, *n* = between 5 and 9 individual channels.

**FIGURE 6.**
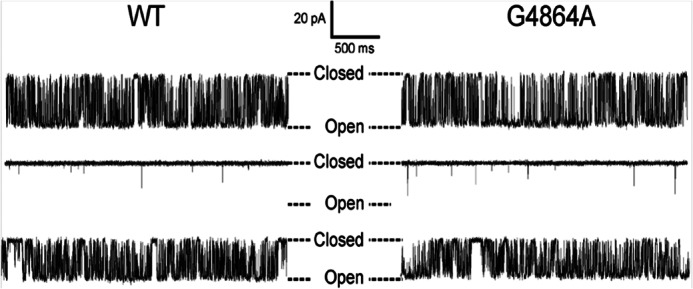
**Variation in WT and G4864A RyR2 open probability with changing cytosolic calcium.** Gating activity of representative WT and G4864A RyR2 channels was recorded in 210 mm KCl at a holding potential of +40 mV. *Upper traces* show gating transitions in 10 μm Ca^2+^ (cytosol and luminal). Open probability is reduced markedly when cytosolic Ca^2+^ is lowered to 0.7 nm following the addition of chelating ligands (*middle traces*). Subsequent elevation of cytosolic Ca^2+^ to 100 μm increases the open probability of both channels (*lower traces*). Luminal Ca^2+^ was buffered at [Ca^2+^] = 50 nm in the *middle* and *lower traces*.

**FIGURE 7.**
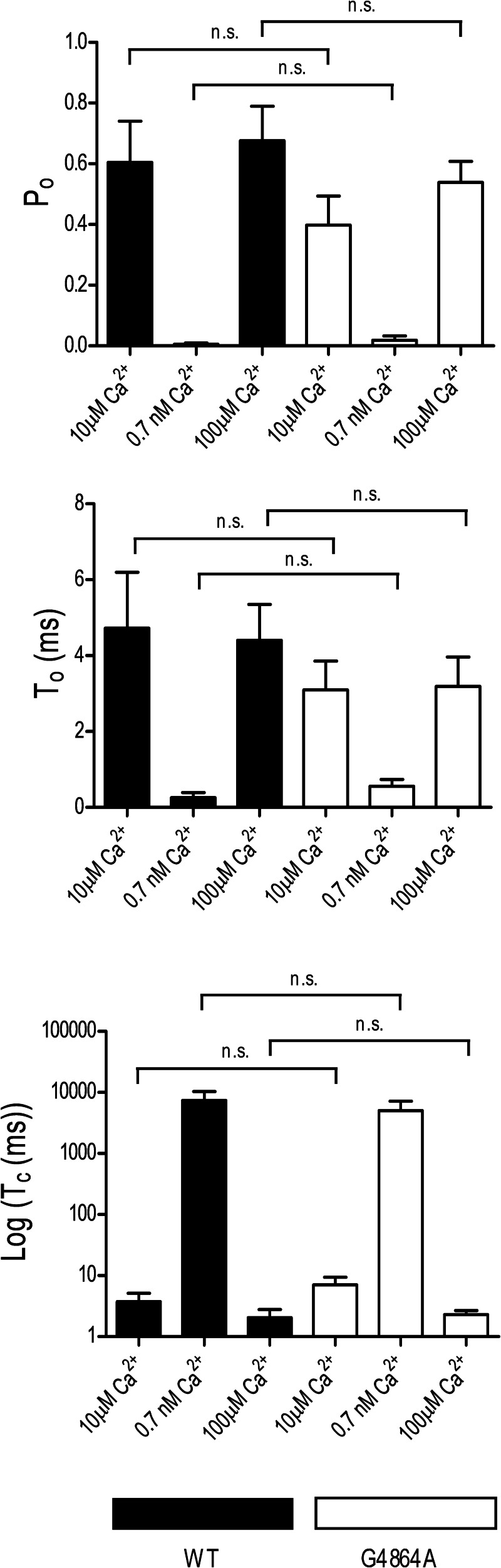
**Variation in mean WT and G4864A RyR2 open probability with changing cytosolic calcium.** Experimental conditions were as described in the legend for Fig. 6. Data are displayed as mean ± S.E. for 5–10 individual channels. An unpaired *t* test was performed and statistical significance is indicated by asterisks. *Upper*, *P*_o_, open probability, *Middle*, *T_o_*: mean open time. *Lower*, *T_c_*, mean closed time. *n.s.*, not significant.

Detailed information on the consequences of residue replacement in RyR2 can be obtained by monitoring the ion handling and gating characteristics of individual channel molecules incorporated into planar phospholipid bilayers under voltage clamp conditions. Consistent with the data obtained in the macroscopic assays described earlier, incorporation of RyR2, in which Gly^4864^ had been replaced with alanine, into bilayers yielded channels with ion handling and gating characteristics equivalent to those of WT RyR2. The plot in Fig. 5 shows the relationship between single channel current amplitude and holding potential for WT and G4864A channels monitored in symmetrical 210 mm KCl. We observed no significant difference in the conductance of the two species of channel (WT; 641 ± 4 picosiemens, G4864A; 637 ± 3 picosiemens). The responses of the WT and G4864A channels to changing cytosolic Ca^2+^ were also equivalent, as demonstrated in Fig. 6. The representative traces, monitored in symmetrical 210 mm KCl at a holding potential of +40 mV, demonstrate a dramatic decrease in single channel open probability (*P*_o_) of both WT and G4864A channels accompanying a reduction in cytosolic Ca^2+^ from 10 μm to 0.7 nm as the result of the addition of the EGTA, HEDTA, and nitrilotriacetic acid chelating mixture. Subsequent elevation of the cytosolic Ca^2+^ to 100 μm raises the *P*_o_ of both species of channel and produces a small decrease in single channel current amplitude as Ca^2+^ competes with K^+^ as the charge-carrying species (32). Analysis of the gating parameters of WT and G4864A channels is shown in Fig. 7 and reveals no significant difference in the variation of WT and G4864A channel *P*_o_ with changing cytosolic Ca^2+^. Similarly, determination of mean open and closed times demonstrates no significant difference in the mechanisms underlying altered *P*_o_ in response to changing cytosolic Ca^2+^ in the WT and G4864A channels.

Caffeine-induced *in situ* release of stored Ca^2+^ and the binding of [^3^H]ryanodine to mixed membrane preparations indicate that RyR2 function is modified by the replacement of Gly^4864^ with either valine or proline. Unlike the situation with WT and G4864A preparations, where channel incorporation into the bilayer was seen routinely following the addition of purified protein in the presence of an osmotic gradient, consistent incorporation of G4864V and G4864P channels was not observed. To maximize the possibility of channel incorporation from these preparations, we devised a protocol in which, following bilayer formation, up to four successive additions of purified channel protein were made at 5-min intervals. This protocol was carried out 28 times with material from three preparations of purified G4864V RyR2, but no incorporations of functional RyR2 channels occurred. This observation, together with an absence of caffeine-induced Ca^2+^ release *in situ* and an inability to bind [^3^H]ryanodine, indicate that G4864V RyR2 channels are unable to open.

**FIGURE 8.**
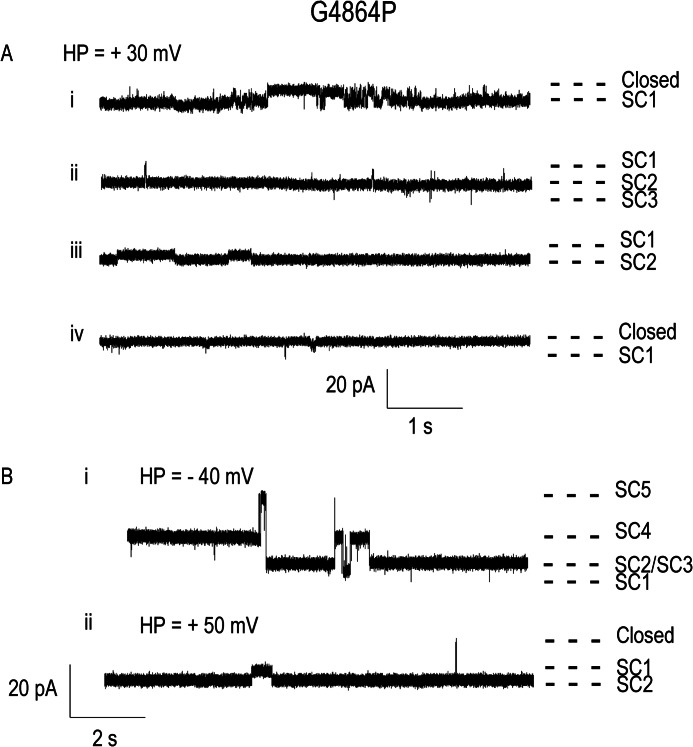
**Representative single channel traces of the G4864P RyR2 channel incorporated into planar lipid bilayers.** *A*, current fluctuations from four different channels recorded at a holding potential (*HP*) of + 30 mV are displayed in the *top panel* (downward deflections represent opening events). *Ai* shows a rare example of long closing events within a period of high *P*_o_. *Aii* and *Aiii* depict the oscillations between various subconductance (*SC*) states without return to the closed state. *Aiv* is the only G4864P channel we found having a very low *P*_o_. *B*, the single channel activity of *Aii* and *Aiii* was further observed at different voltages in *Bi* and *Bii*, respectively. *Bi* shows additional subconductance states observed at −40 mV (upward deflections represent opening events). We cannot establish whether these additional states result from the activity of the same channel or the opening of a second channel. *Bii* shows a rare full closing event. Note the different time base in *panels A* and *B* of this figure.

Using purified channels from three separate transfections, we observed 25 incorporations producing current fluctuations from base line following the addition of G4864P RyR2 to planar bilayers, but in only 10 of these were resolvable gating transitions seen. None of these events had properties characteristic of WT RyR2 function. Rather, as shown in Fig. 8, incorporated channels were predominantly open with fluctuations between different conductance states. Large variations in open state amplitude, variable responses to changes in cytosolic Ca^2+^, and bilayer instability at higher holding potentials meant that it was not possible to carry out a quantitative assessment of single G4864P RyR2 channel function.

## DISCUSSION

Molecular modeling of the PFRs of RyR isoforms has demonstrated that these regions are likely composed of structural elements analogous to those of K^+^ channels (4, 5). The observation of putative equivalent gating components, such as the S4-S5 linker (9) and a G*XXXX*A hinge motif in the pore-lining inner helices (4, 5), suggests that gating in the two species of channel may also involve equivalent mechanisms.

The G*XXXX*A hinge motif originally identified by Jiang *et al.* (11) is present in the majority of K^+^ channels (15), and consistent with the original proposal that gating transitions occur as the result of inner helix flexing at the glycine residue of this motif, mutation of this residue has severe consequences for gating in many K^+^ channels (*e.g. Shaker* (14)). However, in some K^+^ channels, flexing of the inner helix does not require a glycine residue at this position. A proportion of these channels have residues other than glycine at this position (15), and others that do have a glycine retain function when it is replaced by alanine (*e.g.* hERG (17)).

In the current study, we have explored whether, and how, the glycine residue of the G^4864^LIIDA^4869^ motif of RyR2 TM10 contributes to channel gating. To do this, we made selected mutations of glycine 4864 that, based on evidence provided by analogous mutations in K^+^ channels, will reveal the degree of involvement of this residue in RyR2 channel gating.

The resulting data indicate that the contribution of Gly^4864^ to RyR2 gating demonstrates features of both a classical glycine hinge, as typified by Gly^466^ in *Shaker*, and an atypical mechanism exemplified by Gly^648^ in hERG. In *Shaker*, a small increase in residue volume at position 466, as the result of substitution of the hinge glycine by alanine, prevents channel opening (14). In contrast, in both RyR2 and hERG, the ability of the channel to open is unaffected by the substitution of an alanine residue for the putative hinge glycine. In hERG, it has been proposed that alanine substitution at position 648 produces little alteration in gating because this maneuver does not diminish the overall inherent flexibility of the inner helix (17). The equivalence in function of WT and G4864A RyR2 channels suggests that the inner helix of the RyR2 PFR has a similar intrinsic flexibility.

In *Shaker*, substitutions by residues that further increase the volume at position 466 (including valine) also prevent channel opening (14). However, in hERG, a larger increase in the side chain volume at position 648, as the result of replacing glycine with valine (and other large amino acids), produces a significant increase in the probability of the channel being open, most likely because increased bulk at position 648 disrupts the tight packing of the PFR helices (17). Hardman *et al.* (17) also suggest that the discrepancy in the consequences of glycine substitution on open probability in *Shaker* and hERG highlights a significant difference in the inherent conformational stability of these two K^+^ channels, with the closed state more favorable in *Shaker* and the open state more favorable in hERG. RyR2 channels in which Gly^4864^ has been replaced with valine do not release Ca^2+^ in response to caffeine stimulation, do not bind [^3^H]ryanodine, and do not form functional channels on reconstitution into planar bilayers. All of these observations are consistent with the proposal that G4864V RyR2 channels are stabilized in a closed conformation and, following the reasoning set out in Hardman *et al.* (17), that, as is the case for *Shaker* K^+^ channels, the energetically favored gating conformation of the RyR2 PFR is closed.

The introduction of a proline residue will produce a rigid deformation or kink in a pore-lining α-helix, and replacement of putative glycine hinge residues by proline in both *Shaker* (14) and hERG (17) results in an increase in channel *P*_o_. Proline substitution of Gly^466^ in *Shaker* and Gly^648^ in hERG yielded channels that opened in response to activation but once open were reluctant to close. Substitution of proline for Gly^4864^ in RyR2 gave rise to effects that can be interpreted as broadly analogous to those observed in *Shaker* and hERG. Caffeine-induced release of stored Ca^2+^ in HEK cells expressing G4864P RyR2 indicates that at least a proportion of these channels can be stimulated to open and when open provide a pathway for Ca^2+^ to flow down its electrochemical gradient. However, incorporation of purified G4864P RyR2 channels into planar bilayers demonstrates that proline substitution leads to a very significant alteration in channel function. Variable single channel current amplitude and the observation of long, reduced conductance, open events indicates that the mechanisms of both ion translocation and gating are affected, most likely as a consequence of a proline-induced alteration in the conformation and flexibility of the inner helix. Low rates of functional channel incorporation of G4864P RyR2 into bilayers could indicate that proline substitution significantly affects the ability of the channel to open, and the long open times of channels that were functional in the bilayer demonstrate that proline residues at 4864 impede channel closing.

The inability of G4864P RyR2 to bind [^3^H]ryanodine might, at first sight, appear paradoxical given that, in the bilayer, functional channels have a high open probability and that ryanodine binds to the open state of the channel. However, this apparent anomaly could be resolved if the conformational change resulting from proline substitution either limits the access of ryanodine to its binding site or increases the rate at which bound ryanodine dissociates from its site. A precedent for the latter phenomenon exists as we have reported that mutation of the residue adjacent to Gly^4864^ in RyR2 (Gln^4863^) to alanine, although having only a minor effect on the rate of association with the ryanodine binding site, produces a very dramatic increase in the rate of dissociation (33) and abolishes equilibrium binding of [^3^H]ryanodine to membrane vesicles (21).

The current studies provide the first demonstration that gating of the RyR channel is influenced by the properties of the glycine residue in the putative inner helix G*XXXX*A hinge motif. A relatively small increase in residue bulk at position 4864 of RyR2, as the result of replacing the native glycine with alanine, does not alter gating. In contrast, gating is disrupted when glycine 4864 is replaced with the considerably larger valine or the less flexible proline.

The demonstration that gating of RyR2 requires some degree of flexibility at position 4864 in TM10 adds further support to the proposal that the PFR of the RyR channel is composed of structural elements equivalent to those found in K^+^ channels and that these elements are likely to have a similar topology in the two species of channel. However, comparison of the consequences of residue substitution in RyR2 with equivalent substitutions in *Shaker* and hERG K^+^ channels demonstrates that although the helices lining the cytosolic cavities of all three channels contain the G*XXXX*A motif, the mechanisms underlying conformational changes in these helices during gating are considerably different. The data reinforce earlier findings in K^+^ channels that the presence of the G*XXXX*A hinge motif in the inner helices of the PFR does not guarantee that the mechanisms underlying helix flexibility during gating will be the same.

## Footnotes

3 The abbreviations used are:

RyR: ryanodine receptor
hRyR2: human RyR2
PFR: pore-forming region
hERG: human Ether-à-go-go-related gene
HEDTA: *N*-(2-hydroxyethyl)ethylenediaminetriacetic acid
TM10: transmembrane helix 10
eGFP: enhanced green fluorescent protein.
